# Influence of Surface Processing on the Biocompatibility of Titanium

**DOI:** 10.3390/ma4071238

**Published:** 2011-07-06

**Authors:** Kornelia Wirsching, Karla Lehle, Peter Jacob, Otto Gleich, Jürgen Strutz, Pingling Kwok

**Affiliations:** 1 Ear, Nose, and Throat Department, University of Regensburg, Regensburg 93042, Germany; 2 Cardiothoracic Surgery Department, University of Regensburg, Regensburg 93042, Germany; 3 Ear, Nose, and Throat Department, Sørlandet Sykehus, Kristiansand N-4600, Norway

**Keywords:** biocompatibility, Titanium surface processing, Titanium ossicular implant, Kurz

## Abstract

Surface conditioning of titanium middle ear implants results in an improved biocompatibility, which can be characterized by the properties of fibroblasts cultured on conditioned surfaces. Titanium has been established as a favorable biomaterial in ossicular chain reconstruction. The epithelization of the surface of the implants is important for their integration and stable positioning in the middle ear. Mouse fibroblast cells were cultured on platelets made from pure Grade 2 titanium. Platelets that had been etched along their production process were compared to unetched platelets. The DNA in the cell nuclei was stained with DAPI and the actin filaments of the cytoskeleton were stained with FITC-conjugated phalloidin in order to analyze the cells grown on etched and unetched platelets by fluorescence microscopy. SEM (scanning electron microscopic) images were used to compare the surface structure of etched and unetched titanium platelets. There was a statistically significant increase of the area covered by the cytoplasm and increased actin expression by fibroblasts grown on the etched titanium platelets. In addition, the area of the platelets covered by nuclei on the etched platelets exceeded on average the one on unetched platelets, although this difference was not significant. The SEM pictures comparing unetched and etched titanium platelets showed a clear difference in surface structure. Surface conditioning of titanium implants improved the epithelization by fibroblasts and consequently etched titanium should be the preferred biomaterial for reconstructive middle ear surgery.

## 1. Introduction

Finding the perfect material for ossicular chain reconstruction has been a challenging issue for the last 40 years. In 1999 Stupp *et al.* were the first to publish their three years experience with titanium ossicular chain reconstruction [1]. Since then titanium has become the most popular biomaterial in middle ear surgery. High biocompatibility, biostability and excellent sound conduction favor the use of titanium for the reconstruction of the ossicular chain [2,3,4,5]. In ossicular procedures partial or total ossicular replacement prostheses (PORP/TORP) are interposed between the malleus handle or tympanic membrane and the stapes capitulum or footplate. The fenestrated headplate of the prostheses provides sufficient view for a precise positioning of the shaft on the stapes footplate. Important for integration and stabilization in the middle ear, is the covering of the surface of the biomaterial with fibrous tissue and mucosal cells. Titanium possesses properties that support epithelization without foreign-body reaction [6,7]. In the presence of oxygen, titanium is coated with a thin layer of titanium dioxide which prevents tissue modifications, cell mediated hypersensitivity, as well as corrosion of the implant [8]. However, the production process can leave behind chemical and physical particles on the surface of the implant, potentially causing tissue reactions and disintegration of the prosthesis. For the removal of these residuals, all KURZ Medicals (Heinz Kurz GmbH, Dusslingen, Germany) ossicular prostheses are subjected to a complex cleaning process that accounts for 30% of the whole manufacturing time. A major part of this process is the surface conditioning by the use of a special etching technique. This is followed by an examination using Scanning Electron Microscopy (SEM) and energy dispersive X-ray spectroscopy (EDX) of each batch for residual wear particles.

The aim of the present study was to find out if the surface conditioning process of titanium ossicular implants results in an improved biocompatibility measured by the analysis of fibroblasts grown on conditioned surfaces.

## 2. Materials and Methods

### 2.1. Titanium Platelets

**Figure 1:**
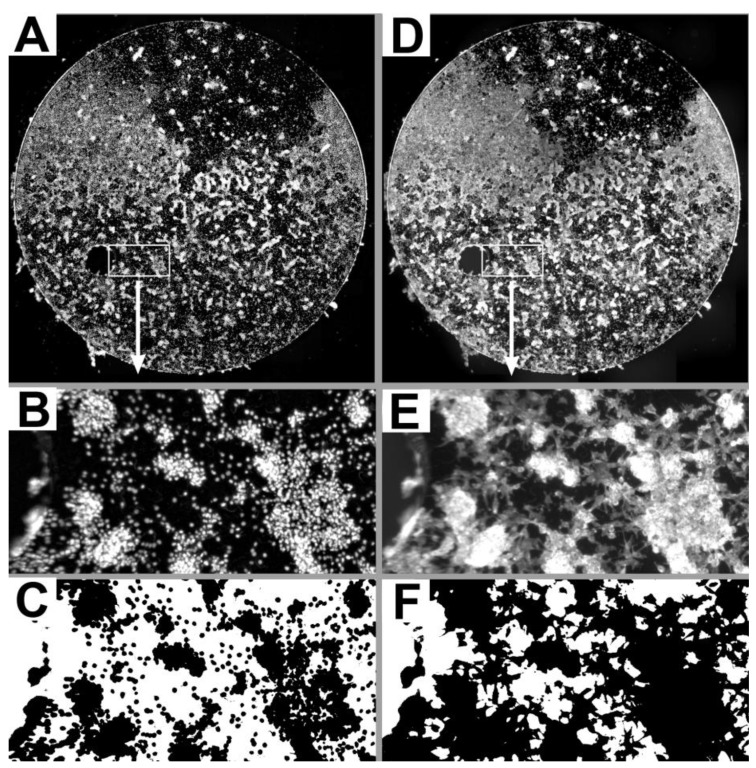
Low power composite images of DAPI stained nuclei (**A**) and FITC-labeled actin (**D**) of one etched titanium platelet (see also Figure 2 F). The boxed 1350 µm* 675 µm regions in the low power overviews are shown at higher magnification for nuclei (**B**) and actin (**E**). In the binary images (**C,F**) corresponding to B and E, pixels with gray levels above the respective gray level threshold (see methods) are shown in black. Using adequate threshold settings DAPI stained nuclei (**C**) and FITC-labeled actin (**F**) were well discriminated from the surface of the platelet not covered by nuclei or cytoplasm (shown in white).

KURZ Medicals provided 24 commercially pure titanium platelets (grade 2/ASTM F 67: Standard for unalloyed Titanium, for Surgical Implant Applications DIN ISO 5832-2) with a diameter of 7.92 mm and a thickness of 250 µm. The platelets were submitted to the KURZ standard cleaning process but etching was omitted from the cleaning process in 12 of the 24 platelets. 16 thereof were used for cell culturing; the remaining 8 platelets were examined under a Scanning Electron Microscope. To allow a more even dissemination of cells as well as an easier analysis of the cell growth under the fluoresescence microscope, the provided platelets had no engravings and only a single notch instead of the typical fenestrations of the headplates (see Figure 1).

### 2.2. Cell Cultures

Frozen mouse fibroblasts (cell line L929 American Type Culture Collection CCL I fibroblast, NCTC clone 929, Manassas, VA, USA) were thawed and cultured in T25 flasks (Easy Flask T25: Nunc Cat. # 156367) in Dulbecco's Modified Eagle’s Medium (DMEM: PAN Cat. # P04-01550) supplemented with 10% v/v fetal bovine serum (FKS: Sigma Cat. # F7524) and 1% v/v Pen Strep (PAA Cat. No. P11-010). After 3–6 days the cells were dissociated with 0.5% Trypsin-EDTA (Sigma Cat. # T4174) in PBS and seeded in DMEM with 10% FKS and Pen Strep at a density of 100,000 cells per well in 6 well plates (multidish 6 well: Nunc Cat. # 140675).

After 2–7 days these cultures were again dissociated with Trypsin-EDTA and seeded in a EC-Medium (Endothel cell growth medium, Promocell Cat. No C-22010, supplemented with 2.44% v/v Supplement pack Promocell Cat. # C-39215, 0.24 mg/mL L-Glutamin Sigma Cat. # G8415, 50 mg/mL Gentamycin Sigma Cat. # G1397, 0.5 mg/mL Amphotericin B Lonza Cat. # Lonz 17-836E and 10% human serum of own production) at a concentration of 50,000 cells per mL. The titanium probes were sterilized with 70% ethanol and after drying they were placed in the wells of cell culture plates (48 well plates/ Nunc Cat. # 150687) and combined with 500 μL of the cell suspension. The cells were cultivated in the incubator at 37 °C with 5% CO_2_ for one week and the medium was changed every two to three days. Pairs of etched and unetched titanium platelets were incubated and processed in parallel. Thus corresponding pairs of etched and unetched platelets were regarded as matched pairs (dependent groups) and a non-parametric paired Wilcoxon test was used for the statistical comparison.

### 2.3. Histologic Preparation

The culture medium was gently removed from the wells and replaced by 4% paraformaldehyde solution in 0.1 M posphate buffer (pH 7.4). After 15 min of fixation under low frequency agitation on a shaker, the fixative was exchanged with fresh fixative and the probes were incubated for another 15 min.

To permeate cells for the phalloidin staining of actin, the fixative was replaced twice for 30 min by 0.3% Triton in 0.1 M phosphate buffer (pH 7.4) and the specimens were agitated. Then the Triton buffer was replaced by 500 μL FITC-conjugated phalloidin solution (Invitrogen, Cat. # F432, 40 μL stock solution diluted in 2 mL 0.1 M phosphate buffer with 0.3% Triton). The wells were placed on the shaker for the actin staining process. After one hour the FITC solution was aspirated and the specimens washed twice for 10 min with phosphate buffer.

Finally, each platelet was placed on a hollow-ground slide and covered with a cover slip using Vectashield with DAPI (Linaris, Cat. # H-1200) in order to stain nuclei. Consequently, the cell nuclei of the fibroblasts on the titanium platelets were stained by DAPI and the actin filaments of the cytoskeleton were labeled by FITC.

### 2.4. Analysis

The specimens were examined under a Leica DM RBE microscope (Leica Mikrosysteme, Bensheim, Germany) with epifluorescence. Images were digitized with a Spot RT3 Slider camera (Diagnostic instruments, Stirling Heights, Mich., USA) and the software VisiView (Visitron Systems GmbH Puchheim, Germany). In order to get an overview of the whole titanium platelet at sufficient resolution for the subsequent analysis, a grid of overlapping pictures of the whole titanium platelet was taken with a Leica PL Fluotar 5×/0.12 lens resulting in a nominal resolution of 1.50 µm/Pixel. All pictures were digitized as 14 bit gray level images using standardized microscope settings and an exposure time of 1000 ms for the DAPI fluorescence filter and 2000 ms for the FITC fluorescence filter. To cover the whole titanium platelet between 18 and 29 overlapping pictures were digitized and a composite of these overlapping pictures was generated using the software package ImageJ 2.44c with the 2D/3D stitching macro from Stephan Preibisch (“http://fly.mpi-cbg.de/~preibisch/stitching.html”). Composite images of FITC stained actin were assembled without further processing of the individual pictures. To improve contrast, each picture of DAPI stained nuclei was subjected to background subtraction using a “rolling ball radius” of 50 pixels before assembling the composite image. Figure 1 shows examples of DAPI stained nuclei (A) and FITC-labeled actin (D) for one etched titanium platelet as an overview at low magnification and selected regions at higher magnification (B, E).

For the quantitative analysis of the cell coverage of the titanium platelets the images were opened with ImageJ. By choosing an adequate threshold value cytoskeletal elements stained by the FITC conjugated phalloidin and DAPI stained cell nuclei were discriminated from the surface of the titanium platelets not covered by nuclei or cytoplasm of the fibroblasts (Figure 1C, F). Thresholds were determined empirically and set to a gray level value of 200 for DAPI stained nuclei and 1000 for FITC labeled actin and these settings were used for the analysis of all composite images. For each titanium platelet the number of pixels with a gray level above threshold was determined. Using an adequate calibration the area of the platelet covered by supra-threshold pixels was calculated. This area was then expressed as percentage of the total platelet surface area that was used for subsequent comparisons. In addition, the mean gray level of the pixels with gray levels above threshold was determined as a measure of staining intensity.

The software SPSS for Windows PASW Statistics 17.0.2 was used for the statistical analysis.

### 2.5. Scanning Electron Microscopy (SEM)

8 titanium platelets (4 etched *vs.* 4 unetched) were examined by Scanning Electron Microscopy (Cambridge Stereoscan 420; Co. Carl Zeiss NTS GmbH, Oberkochen, Germany) without previous cell cultivation. The platelets were mounted on SEM-stubs using adhesive Conductive-Tabs (Fa. BALTIC Präparation, Koppelheck 34b, 24359 Niesgrau, Germany) and then examined under vacuum with an acceleration voltage of 10 kV. Digital pictures of every probe were taken with a magnification of up to 1000×, displayed and stored with SCAN, Digital Image Processing System 2.1 (Co. point electronic GmbH, Ackerweg 104, 06103 Halle, Germany).

## 3. Results

### 3.1. Qualitative Analysis of Fibroblasts Growth

**Figure 2:**
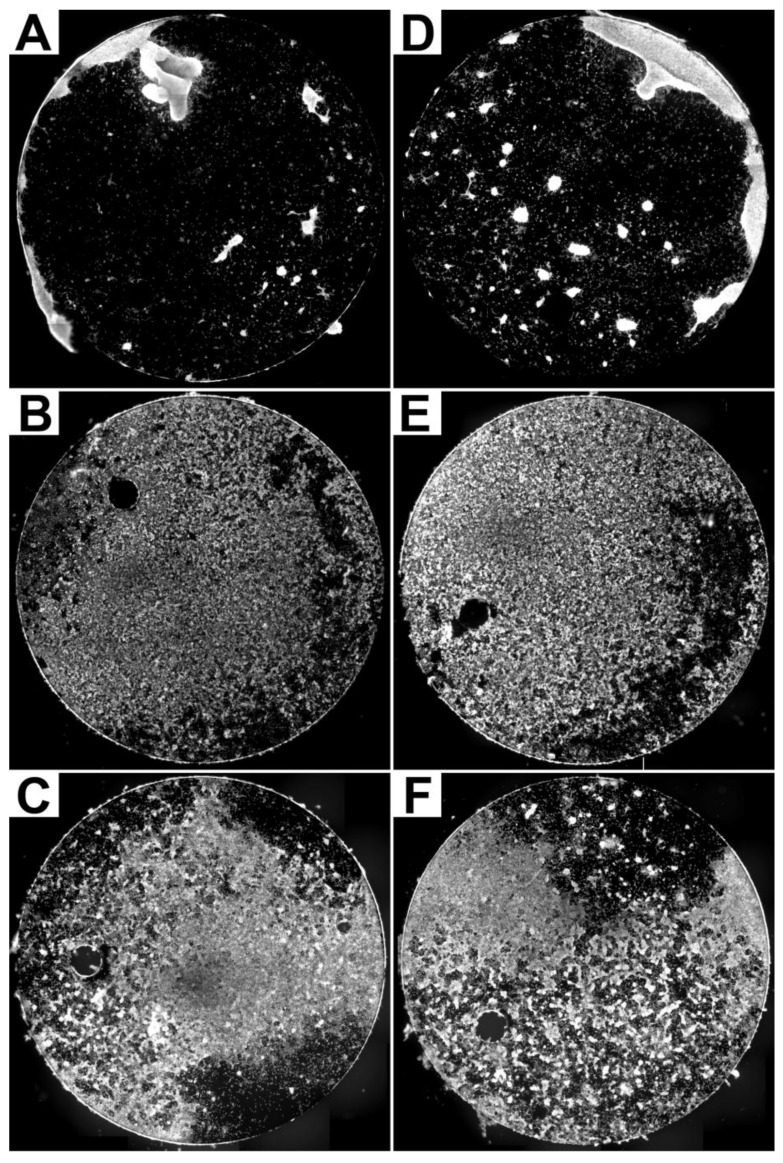
Examples of composite images of pairs (**A**/**D**, **B**/**E**, **C**/**F**) of unetched (**A**, **B**, **C**) and etched (**D**, **E**, **F**) titanium platelets where the actin of fibroblasts has been labelled by FITC-conjugated phalloidin.

First, we compared the fluorescence microscopic images of the fibroblasts grown on etched and unetched titanium platelets qualitatively by visual inspection. Growth of fibroblasts was present on all etched and unetched titanium platelets although the proportion of the platelet surface covered by fibroblasts varied substantially between experiments (Figure 2).

The pattern for the distribution of FITC-labeled actin and DAPI stained nuclei of the fibroblasts grown on the platelets was similar when viewed at low magnification (compare Figure 1A and D). Differences due to the fact that the nucleus is a cell organelle within the cytoplasm became obvious at higher magnifications (compare Figure 1B and E).

The examples illustrated in Figure 2 demonstrate that a qualitative visual comparison was not sufficient to clearly identify an obvious difference of fibroblast growth on the unetched and etched platelets. Thus a quantitative image analysis approach was used in our search for a potential effect of platelet conditioning on cultured fibroblasts.

### 3.2. Quantitative Analysis of Platelet Surface Covered by Cytoplasm and Nuclei

One measure to compare fibroblasts grown on unetched and etched titanium platelets is the proportion of the platelet surface covered by cytoplasm and nuclei that is listed in Table 1 for the different experiments and experimental conditions.

**Table 1:** The proportion of the platelet surface area in percent covered by cytoplasm (as indicated by FITC-labelled actin) and nuclei (as indicated by DAPI stained nuclear DNA) for unetched and etched titanium platelets.

Experiment	FITC unetched	FITC etched	DAPI unetched	DAPI etched
1	11.45	31.36	3.45	12.05
2	13.15	19.98	8.46	14.07
3	17.85	19.84	11.47	9.22
4	38.88	39.76	13.14	13.48
5	52.41	66.38	55.33	58.03
6	59.04	63.43	42.25	44.83
7	71.12	66.85	50.07	47.06
8	79.37	94.18	25.77	35.24
Mean +/− Stdv.	**42.91 +/− 24.99**	**50.22 +/− 24.87**	**26.24 +/− 19.03**	**29.25 +/− 18.03**

The quantitative analysis presented in Table 1 confirms a considerable degree of variation between the different experiments. Consistent with the fact that the nucleus is only an organelle while the cytoplasm represents the whole cell, the proportion of the platelets covered by nuclei is consistently lower than that covered by the cytoplasm (see also Figure 1C, F).

The data show that in 7 of the 8 experiments a higher proportion of the surface of etched platelets was covered by fibroblast cytoplasm as compared to unetched platelets. On average 42.91% of the area was covered by the cytoplasm of fibroblasts on unetched compared to 50.22% on etched platelets.

This corresponds to a 17% increase on etched as compared to unetched platelets. The statistical comparison of the data from the pairs of unetched and etched platelets using a Wilcoxon test revealed that this difference was significant (*p* = 0.036).

The quantitative analysis also showed that on average nuclei of fibroblasts grown on etched platelets covered a larger proportion of the surface (29.25%) as compared to unetched platelets (26.24%). This represents an increase by 11%, but the difference was not significant (*p* = 0.123).

### 3.3. Quantitative Analysis of Staining Intensity

The gray level is a measure of fluorescence or staining intensity respectively. The analysis of gray level or staining intensity is a way to compare the amount of FITC labeled actin or DAPI labeled DNA of fibroblasts grown on differently treated titanium platelets. The mean gray level of the supra-threshold pixels from the FITC and DAPI composite images of fibroblasts grown on unetched and etched platelets are listed in Table 2.

The comparison of the mean FITC gray level of the cytoplasm from fibroblasts grown on unetched and etched titanium platelets demonstrates in 7 out of the 8 experiments higher values for etched (mean 2408) as compared to unetched (mean 2192) platelets. The statistical comparison (Wilcoxon test) confirmed that this difference in gray level was significant (*p* = 0.017). The higher gray levels in the cytoplasm of fibroblasts grown on etched platelets indicate that they contain more actin as compared to fibroblasts grown on unetched titanium platelets.

**Table 2:** The mean gray level of supra-threshold pixels of the cytoplasm (as indicated by FITC-labeled actin) and nuclei (as indicated by DAPI stained nuclei) for unetched and etched titanium platelets.

Experiment	FITC unetched	FITC etched	DAPI unetched	DAPI etched
1	2605	2959	488	417
2	2639	2762	425	362
3	2588	2840	490	526
4	1651	1853	369	431
5	2558	2552	353	353
6	1743	2129	577	730
7	2086	2128	875	991
8	1666	2042	846	839
Mean +/− Stdv.	**2192 +/− 425**	**2408 +/− 393**	**553 +/− 190**	**581 +/− 226**

Comparing the gray levels of DAPI stained nuclei of fibroblasts grown on the two types of titanium platelets showed no systematic difference. In 4 experiments mean gray level was higher for the etched as compared to the unetched, in one experiment gray level was the same and in 3 experiments the gray level was higher for the unetched as compared to the etched condition. The statistical comparison by a Wilcoxon test revealed no significant difference (*p* = 0.499). The similarity of the DAPI gray levels indicates that the DNA content of fibroblast nuclei grown on etched and those grown on unetched titanium platelets did not differ.

### 3.4. SEM

**Figure 3:**
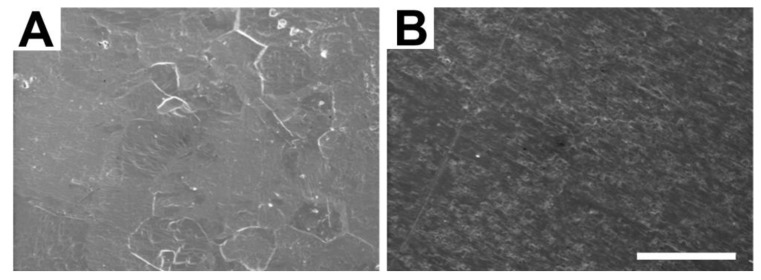
Examples of high power SEM images from the surface of titanium platelets that were either etched (**A**), or not etched (**B**). The white bar represents a length of 50 µm.

All 4 examined unetched platelets showed a rough and grainy surface with scratches and parallel grooves (e.g., Figure 3B) as a result of the manufacturing process. Compared to the unetched condition, etching clearly affected the surface of all 4 analyzed titanium platelets in a similar way (e.g., Figure 3A). In etched platelets, the surface appeared smoother and less rough or grainy resembling a relief map with indentations and elevations. The grooves and scratches due to the mechanical surface processing disappeared on the surface of etched platelets (A).

## 4. Discussion

Titanium has been examined as a material for ossicular replacement with favorable results in human middle ear surgery [2,3,4,5,9]. It is characterized as an excellently well tolerated biomaterial with very low disintegration rates and good sound conduction. Grade 2 pure titanium, which is the raw material for KURZ middle ear protheses, has a Ferrite content of 0.3% ensuring high stability (KURZ Medicals). Remarkable is the low weight of the material (specific weight titanium 4.5 g/cm^3^ *vs*. gold 19.3 g/cm^3^). In general, titanium is a material with a high biocompatibility which facilitates cellular overgrowth [10]. The application in an open implant area like the middle ear with potential germ colonization requires excellent biocompatibility. An important histological criterion for judging the biocompatibility of alloplastic material is the amount of surrounding fibrous tissue after implantation [11]. The mucosal injury during insertion of the prosthesis is a strong growth stimulus. Activated local growth factors support the outgrowth of fibroblasts and epithelial cells. Furthermore extracellular proteins like albumin are well adsorbed by the titanium surface and strongly bound. This is the essential condition for the long term integration of the biomaterial in living tissue [12]. Furthermore, the affinity of titanium towards bone, known as osseointegration [13], leads to a stable fixation between the titanium prosthesis and the stapes footplate. Recent studies have tried to induce this process with osteoinductive substances covering the titanium stapes footplate [14].

Interestingly, by using titanium middle ear implants, revision procedures are necessary in 4.8% [1] (second look procedures excluded) up to 8% [15]. Main reason for revision surgery is the dysfunction of the Eustachian tube causing recurring chronic inflammation and scarring. Stupp *et al* stated a conductive hearing loss due to a too short implant or implant dislocation as another frequent reason for revision surgery [1].

Possible negative influences on the biocompatibility of titanium middle ear implants are manufacturing residues or an unsuitable surface structure of the prosthesis. One hypothesis is that wear particles or debris can lead to cell mediated hypersensitivity and inflammation. This limits the cell overgrowth with fibrous tissue. In the present study we show, that after surface conditioning of titanium platelets the amount of surface covered with fibroblasts increased by 17%. In addition, the actin content, as determined by FITC gray levels, was significantly increased in fibroblasts grown on etched as compared to those grown on unetched titanium platelets. On average, the area covered by nuclei of fibroblasts grown on etched platelets was also higher compared to unetched platelets, but this difference was not significant in the present sample. The difference of fibroblasts grown on etched and unetched titanium platelets correlates with changes of the surface structure induced by the etching procedure (Figure 3).

## 5. Conclusions

In conclusion, our present investigation demonstrates that special processing of titanium middle ear implants leads to increased actin expression and increased coverage by fibroblasts. The cell growth on the prosthesis is a main indicator of its biocompatibility. Furthermore, cell growth supports the stable integration of the implant within the reconstructed ossicular chain. The effects observed in the present study suggest that this type of surface modification may be beneficial for integration in titanium middle ear prostheses. For reconstructive middle ear surgery accordingly processed titanium implants should be preferred.
